# Descriptor engineering in machine learning regression of electronic structure properties for 2D materials

**DOI:** 10.1038/s41598-023-31928-7

**Published:** 2023-04-03

**Authors:** Minh Tuan Dau, Mohamed Al Khalfioui, Adrien Michon, Antoine Reserbat-Plantey, Stéphane Vézian, Philippe Boucaud

**Affiliations:** Université Côte d’Azur, CNRS, CRHEA, rue Bernard Grégory, 06560 Valbonne, France

**Keywords:** Theory and computation, Electronic properties and materials

## Abstract

We build new material descriptors to predict the band gap and the work function of 2D materials by tree-based machine-learning models. The descriptor’s construction is based on vectorizing property matrices and on empirical property function, leading to mixing features that require low-resource computations. Combined with database-based features, the mixing features significantly improve the training and prediction of the models. We find R$^{2}$ greater than 0.9 and mean absolute errors (MAE) smaller than 0.23 eV both for the training and prediction. The highest R$^{2}$ of 0.95, 0.98 and the smallest MAE of 0.16 eV and 0.10 eV were obtained by using extreme gradient boosting for the bandgap and work-function predictions, respectively. These metrics were greatly improved as compared to those of database features-based predictions. We also find that the hybrid features slightly reduce the overfitting despite a small scale of the dataset. The relevance of the descriptor-based method was assessed by predicting and comparing the electronic properties of several 2D materials belonging to new classes (oxides, nitrides, carbides) with those of conventional computations. Our work provides a guideline to efficiently engineer descriptors by using vectorized property matrices and hybrid features for predicting 2D materials properties *via* ensemble models.

## Introduction

Data is one of the core rudiments that feeds artificial intelligence (AI) algorithms for training and data-driven inference. In the field of materials science, many efforts for discovering novel functional materials and their properties with help of the power of data and AI models have been intensely dedicated, providing alternatives to classical computation techniques such as work-horse computational and quantum chemistry methods^1,2^. As a matter of fact, the data-driven technique permits to mitigate impediments of the classical methods which require huge computational resources, time consumption and complexity of simulated physics. For instance, Pimachev *et al.*^3^ pointed out that the time consumption for Density Functional Theory (DFT) calculations scales quadratically while feature extraction for model training scales linearly with system size. For a same system size, one would need few hours to have comparable results with AI algorithms (feature extraction, training data and prediction) against 10$^{3}$ CPU hours of DFT calculations. Using machine learning (ML) to predict the band gap of materials, with or without combining DFT calculations is a notable illustration of feasibility and effectiveness of ML in the prediction of materials properties^4–6^. Efforts to develop algorithms in ML which are suitable to predict specific physical properties were also addressed^7–9^. These works have shown the power of ML in terms of theoretical characterization of materials.

The application of data science to bi-dimensional (2D) materials has been boosted by the quest of new classes of 2D materials since the discovery of graphene, the first 2D monolayer^10^. The milestones in the development of 2D materials like the experimental discovery of new synthetic 2D materials^11^ would be speeded up thanks to AI assistance^12–14^. In the same perspective, the quest of 2D materials possessing high-temperature ferromagnetic order at long range has been also subject to data-driven studies for rapid discovery of stable magnetic 2D materials^15^. In another physics picture, defects in 2D lattices of transition metal dichalcogenides (TMDs) or hexagonal boron nitride (h-BN) are of particular interest because they might play a prime role as single emitters in the quantum emission process^16,17^ and non-volatile resistance switching^18^. ML-assisted design of point defects in 2D materials has been proposed by using a transfer learning procedure, then scoring the defect structures in order to predict ideal 2D candidates hosting defects for these specific applications^19^. Recently, the inference of 2D materials band gap, which is indicative of electronic structures and band offset in 2D heterostructures, is a relevant example regarding the use of ML in the characterization of intelligent data cycles^20,21^.

Besides algorithms employed to predict these properties, the descriptor engineering and feature selection are pivotal factors for reducing the error loss in the training process and for improving the prediction. Universal descriptors based on a set of feature vectors, which is constructed by structural inputs, have shown a good estimation of important properties^22^. However, such a method working solely with specific features could touch limit of physics description when downsizing the dataset size. Up to date, the dataset size of 2D materials is in general small since theoretical and experimental data regarding 2D materials are scarce. A graph-based multilayer descriptor was introduced to discover new magnetic 2D materials endowing stable magnetic ordering^23^. The descriptors were built based on multilayer-based crystal graph that induces a set of more than 230 features even after having used pooling technique to reduce the dimension. Although the results are promising, the approach seems to be tedious because of involvement of high number of features and expensive calculations.

Here, we make use of Computational 2D Materials Database (C2DB) dataset of about 4000 entries, which is reliable data based on DFT calculations, to introduce novel descriptors for boosting the prediction of the electronic structure properties of 2D materials^24,25^. Complementary to the study conducted by Zhang *et al.*^20^ where the models (R$^{2}>$ 90%) are strongly biased (the feature “gap-nosoc” values are too close to the predicted values), our approach consists of introducing descriptors vectorized from property matrices for ML cycles. The descriptors are simple to compute and directly related to electronic properties. We combined these descriptors with empirical function-based descriptor (electronegativity)^26^ to strengthen the efficiency and the stability of the descriptors against the change of target properties and models. Such a combination permits to considerably reduce the dimension of the input features and results in a high performance of models as compared to previous studies. Furthermore, the proposed descriptor processing is simple to implement, robust and it requires low-computation cost.

The efficiency of the constructed descriptors was evaluated through the performance of the ML cycles where the accuracy (R$^{2}>$ 90%, mean absolute error (MAE) down to 0.1 eV) is much higher than the performance obtained with database-based features. Our results are comparable to those of ML cycles with DFT-generated features^20^. Moreover, we have found that the hybrid descriptors or features contribute to reduce the overfitting in the prediction of the work function. Indeed, the discrepancy of MAE metrics for the training set and the test set were decreased by 0.1 eV. Our findings point to consistent relationship of the descriptors with electronic structure properties of 2D materials. Apart from the classical construction of empirical descriptor (electronegativity), the introduction of vectorized descriptors indicates that one could “skip” certain expensive-calculated features which are sometimes biasing sources of the outcomes^20^. Therefore, conventional ML cycles could get decent and comparable results although the volume of features is much less than other approaches^23^. Our findings clearly indicate the breakthrough of the descriptor engineering based on vectorizing, empirical function-based descriptors and domain knowledges in the data-driven prediction of 2D materials properties. Finally, the inference of the band gap and the work function of recently synthesized 2D materials and undiscovered ones (oxides, nitride, transition metal carbides) was carried out to underline the relevance of the descriptor-tailoring method. We could correlate the obtained values with those of *ab initio* calculations and forecast that the unknown work functions of $\hbox {Mo}_{2} \hbox {Ga}_{2}$C and $\hbox {V}_{2} \hbox {Ga}_{2}$C are 4.9 eV and 4.6 eV, respectively.

## Methodology

### Additional descriptor construction

To do so, we have selected relevant atom-atom interactions based on elemental parameters to generate molecule-level descriptors that closely describe their impact on target properties. We use in this study 8 descriptors in total. Four descriptors (number of atoms, cell volume, molecular mass and formation heat) are picked up from the C2DB database. Four additional descriptors were created according the following procedure. One descriptor (electronegativity) was calculated by using empirical equation^26^. For instance, the electronegativity of a molecular formula A$_{a} \hbox {B}_{b}$C$_{c}$ is expressed as $\chi = (A^{a}B^{b}C^{c})^{1/(a+b+c)}$. The three remaining descriptors being vectorized descriptors (covalent radius, dipole polarizability, ionization energy) were built based on atomic property data^27^. Indeed, the band gap and the work function are inherent properties of materials which are fundamentals for featuring their electronic structure. At molecule level, the elemental properties of constituents and the intra-molecular interactions are indicative of how electronic structure evolves. The construction of the vectorized descriptors consists of computing vector-based properties of a molecule derived from property matrices which should have a strong physico-chemical relationship with target properties. The property $\hbox {P}_{i}$ of a molecule is represented by a property matrix those values are filled by the atom-atom pair contribution: $P_{i} = [a_{ij}]_{i,j = 1}^{n} = [{\hat{H}}(ij)]_{i,j=1}^{n}$, where ${\hat{H}}$ is a predefined operator applying on atoms i, j and n is the number of atoms in the reduced stoichiometric formula of the molecule. Depending on the nature of the property, the operator employed in the construction process may be addition, subtraction (absolute value) or multiplication. For instance, a dipole polarizability of an atom-atom pair results from a summation of individual polarizabilities. This operation allows to take into account the atom-atom pair interaction in the molecule. In this study, the choice of the properties (covalent radius, dipole polarizability, ionization energy) was based not only on the relationship with the electronic structure of molecules but also on the non-complexity of calculations to efficiently assess the prediction performance of ML models for low-cost computations. Indeed, by employing these descriptors as input features, we get access to the low levels of the intra-molecular interactions which help the ML solvers to determine resulting electronic properties of molecules. Compared to classical and sophisticated approaches enabling to compute a whole picture of the energy distribution of states in momentum space, descriptor engineering-based ML set-up is fully accessible for low-cost computations. For the sake of simplicity, we adopt a reduced stoichiometry of molecular formula to build the property matrices.

**Figure 1:**
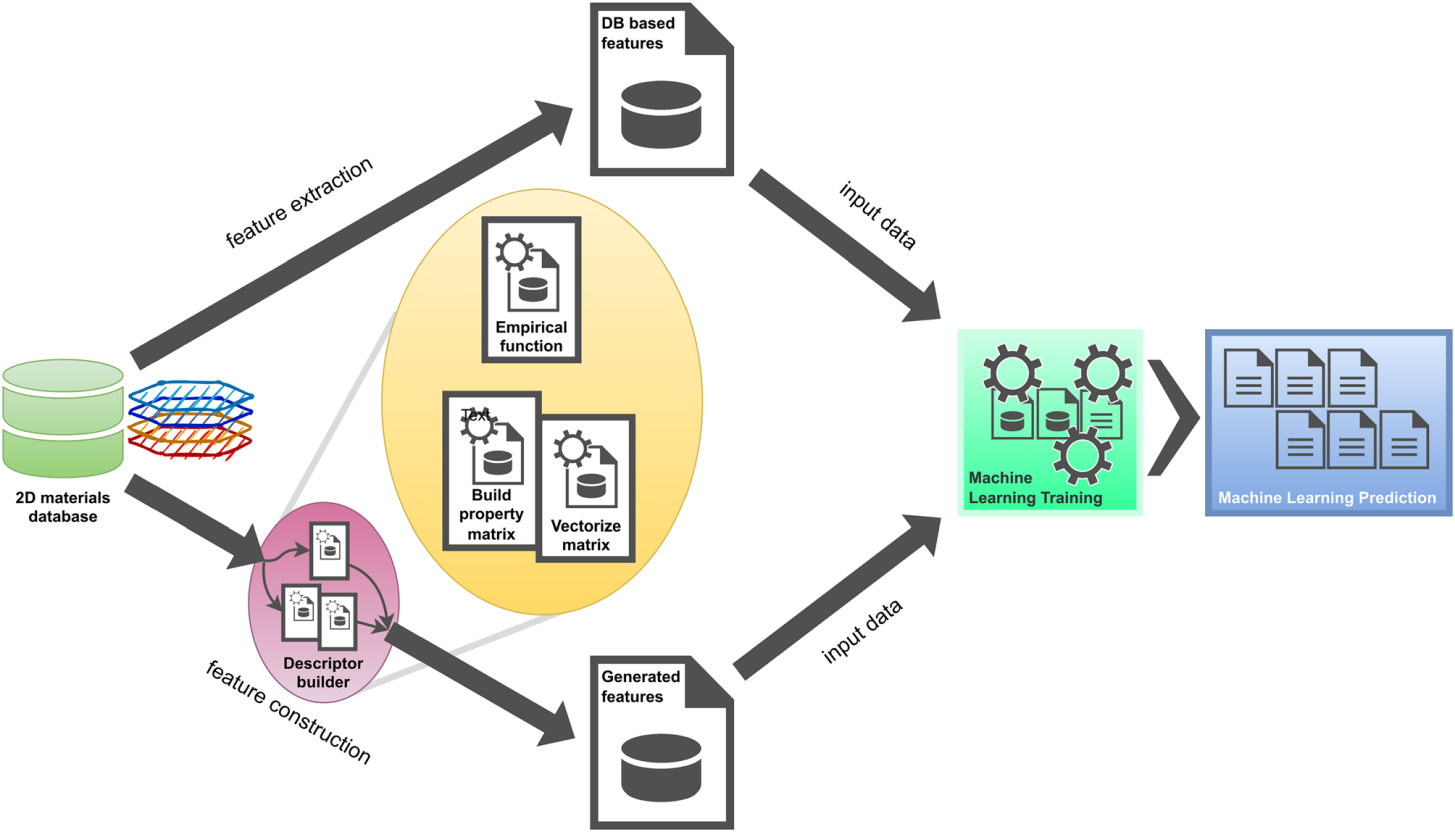
Workflow of the concept of hybrid descriptor engineering: C2DB database feeds the descriptor processing that produces input features for ML training and prediction. The generated features are mixed features from vectorized property matrices and empirical function property.

The next step is to compute property vectors based on the property matrices. We define a property vector as a set of eigenvalues that characterize the property matrix : $P_{i} X = \lambda X$ , where $\lambda$ stands for eigenvalue corresponding to eigenvector X. As the property matrices are symmetric, the eigenvalues get real values. A eigenvalue-based property vector represents characteristic spectrum of a property feature. The purpose of this conversion is twofold: (i) flatten the property matrices, thus greatly reduce the volume of input data for the training process by keeping physics meaning since the eigenvalues, i.e. energy states, are unique for each property matrix; (ii) provide evidence for an efficiency of this hybrid approach, i.e. combination of the ready-to-use features and the simply-built features regarding the property-prediction performance on the limited size of the dataset (around 4000 entries for C2DB). Since the molecule lengths (number of atoms) are different, for the sake of data and input consistency, we impute the missing data of short property vectors with zero eigenvalue (zero state) to achieve the longest molecular length. Figure 1 describes the scheme of data flow that shows the DB, descriptor builder, model solvers and output inference.

### Model selection and machine learning

Random Forest (RF), Gradient Boosting (GB), Extreme Gradient Boosting (XGB) were selected to perform the inference on the band gap and the work function of 2D compounds in the C2DB dataset (see Supplementary Information for basic explanation of the models). These algorithms are tree-based models, in particular gradient boosting algorithms which are well suitable for properties classification and regression as compared to other algorithms^28^. The dataset was randomly split into training and test sets with a ratio of 4:1, respectively. For each ML model, the training was carried out by tuning hyper-parameters with help of grid search (see Supplementary Information) in scikit-learn^29^ and python 3.8 environment. The search permits to select the best model based on the best score (lowest function loss) for a given training set and given ensemble of features. We also employed the cross validation for training and prediction to reduce the randomness effect due to the dataset splitting and the overfitting of the data. The cross-validation sampling is generated by k-fold cross validator for a regression estimator. The selected models showed stable outcome scores regarding the random splits.

## Results and discussion

The outcome evaluation is based on the feature selection: 4 database-based features as reference (feature set 1) and 8 hybrid features (feature set 2, composed of the set 1 and 4 additional constructed features). The details of the features for model training are given in table S1 (Supplementary Information). The first set of features is available in C2DB DB and is intuitively relevant to the target properties. These features are basically related to chemical and crystalline structures. We note that since the dataset and the models are pre-defined, less the number of DB features is, more the generated features-based ML assessment of inference is convincing and generalizing. The comparison with DB features is to show how our descriptor engineering could be simple but as efficient as complex descriptors for a same prediction purpose. To create the second set of features (set 2), we built 4 additional features by following the method described in previous section, then, by merging them with the first set of features. This results in hybrid inputs for a full evaluation of our approach of descriptor engineering.

### Band gap

**Figure 2:**
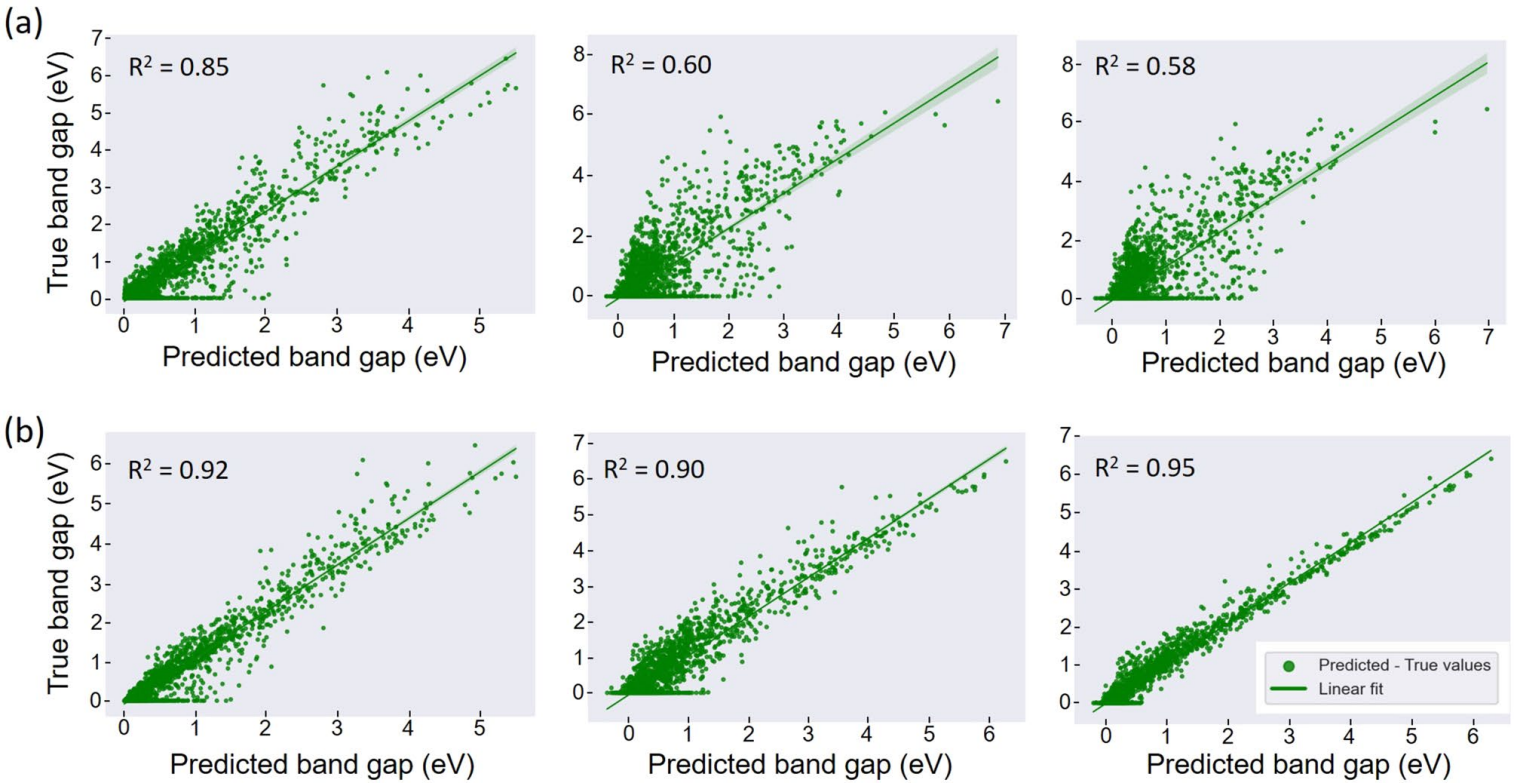
Visualization of the predicted and true values of the band gap by using the feature set 1 (**a**) and the feature set 2 (**b**) for RF, GB, XGB models. The values of R$^{2}$ are also shown. The lines shown with confidence interval of 95% are linear regression of the data points and aid as a guide to the eye.

**Figure 3:**
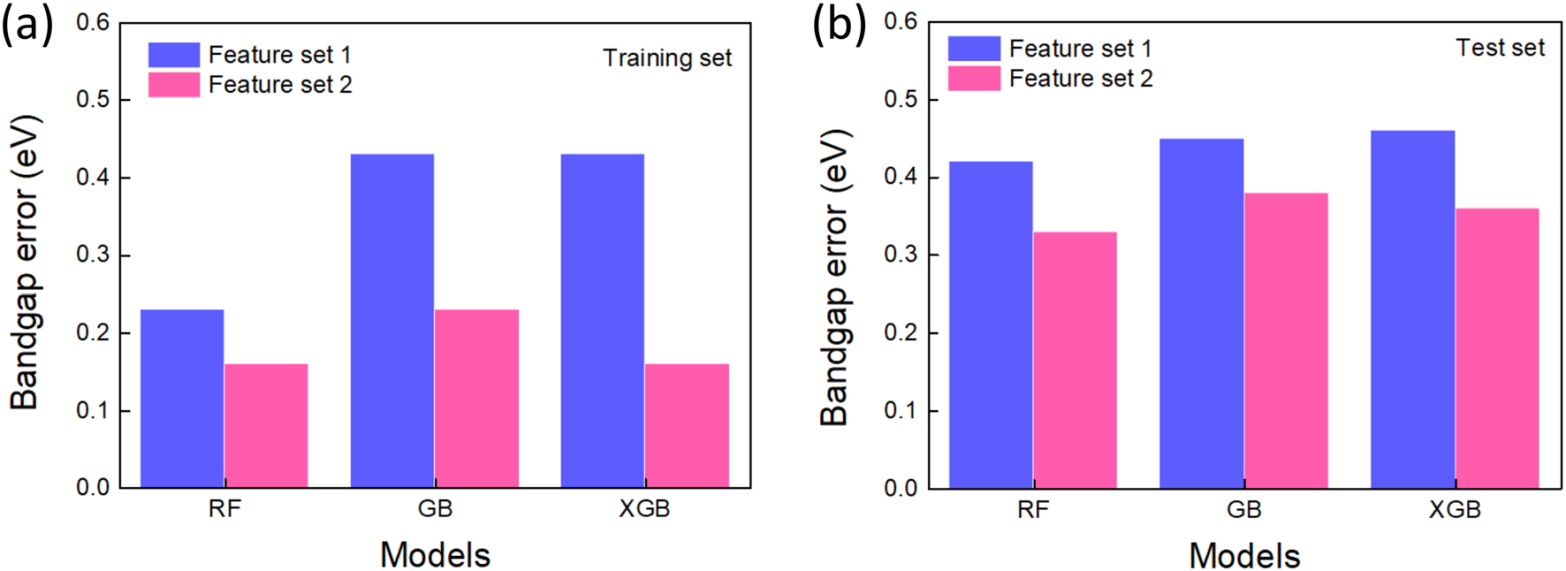
Bar plots of the MAE errors generated by the feature sets 1 and 2. The results are grouped into the training set (**a**) and the test set (**b**) to highlight the out-performance of the ML inference when using the additional features (set 2).

With the feature set 1, we find that the scores of model training set are quite low: R$^{2}>$ 0.58 and MAE of about 0.2–0.4 eV (Figs. 2a, 3a). It turns out that the feature set 1 does not reflect well the inherent electronic properties of the molecules, thus, they poorly help the training, resulting in fair scores. Figure 2a shows the goodness of fit describing the predicted band gap against the true band gap for RF, GB and XGB models with corresponding R$^{2}$ values. A strong correlation is found for RF. We note that the RF model gave R$^{2}$ = 0.85 which is the best score for this set of features. Since the RF regressor outputs an average predicted value generated by the ensemble of trees, such a process greatly reduces the error in a regression process, permitting the model to “ignore” the data fluctuations. The model could capture significant trend of the bandgap variation although this set of features is not much sensitive to the bandgap variation. The MAE score is much lower (50%) for the RF regressor compared to those of two other models (Fig. 3a). The performance of the models on the test set is quantitatively assessed with the help of MAE metrics (Fig. 3b). The MAE value is of 0.42 eV for RF whereas GB and XGB predict the unseen dataset with an error of about 0.45 and 0.46 eV, respectively. The slight difference in MAE values (training versus test sets) is indicative of an overfitting effect for the RF model. The overfitting phenomenon generally occurs when the amount of data is small and there exists a certain inconsistency of data. Apart from this common issue, we raise two major specific reasons regarding our ML process: tiny number of features (set 1) which comes from only 4 descriptors available in C2DB DB; inappropriate tree-related hyperparameters (depth of trees, *etc*.) would bias the fitting mechanism of RF algorithm, thus, it fits noise rather than signal^30^. For the case of GB-based models, an underfitting occurs due to very low values of R$^{2}$ and high errors for both training and test sets. The simplicity of input features may be not compatible with the complexity of gradient-assisted tree growth, resulting in a high bias and a high variance.

The performance of the model training was greatly improved when we embedded the vector features as shown in Figs. 2b and 3. Accordingly, the training metrics R$^{2}$ of all models attain about 0.9 and beyond, especially the highest value of 0.95 for XGB model with exceptional MAE of 0.16 eV. The correlation between the predicted values and true values becomes stronger for both three models as the data cloud is regularly distributed (Fig. 2b). The MAE errors calculated for the test set were also greatly improved, around 0.35 eV for all models, indicating a decent performance of the models over the test set (Fig. 3b). It’s worth noting that the error generated by ML for C2DB dataset is below the error range of experiments versus theoretical simulations which is in general between 0.2 and 0.4 eV^31^. These findings clearly suggest that adding constructed features is beneficial for the training process and the model could catch the data tendency. This also indicates a strong relationship between the constructed vector features and the predicted band gap of 2D materials. Indeed, the selected vector features derived from the property matrices present physico-chemical relationship with respect to the electronic structure of molecules, resulting in a certain degree of dependence of the band gap on those features. Furthermore, a great increase of the scores for GB-based models (GB and XGB) as compared to the one of RF model when including newly designed features reveals an out-performance of descriptor engineering regarding training mechanism. The addition of subsequent trees built in GB-based models is more sensitive to the new features, allowing to optimize the loss function of the predicted band gap upon the training whereas the ensemble building of trees in RF predictor moderately benefits from the additional features. Therefore, these findings point out that the characteristics of a given algorithm hold an important role in the descriptor engineering besides physico-chemical relationship with predicted properties. When the factors (additional features and appropriate model) emerge together, the model performance evaluated *via* training and prediction processes becomes optimal. We also note that the suppression of the underfitting when embedding additional features for GB-based models associated with an overfitting (about 0.2 eV) is still a good compromise for the bandgap prediction. The results allow to validate our approach of data processing and descriptor engineering that are simple to handle, low-computation cost and efficient in terms of model performance.

### Work function

We now make use of the concept of generated features to predict work function which is also an important property of the electronic structure. The work function is a key parameter enabling to assess electrical contacts in 2D materials with metallic electrodes and the band alignment in 2D heterostructures. These concerns are of high importance for the 2D-materials-based applications in electronics, photovoltaics and photodetectors^32,33^. A rapid, low-resource and accurate prediction of such a property would be of high benefit to speed up experimental studies.

**Figure 4:**
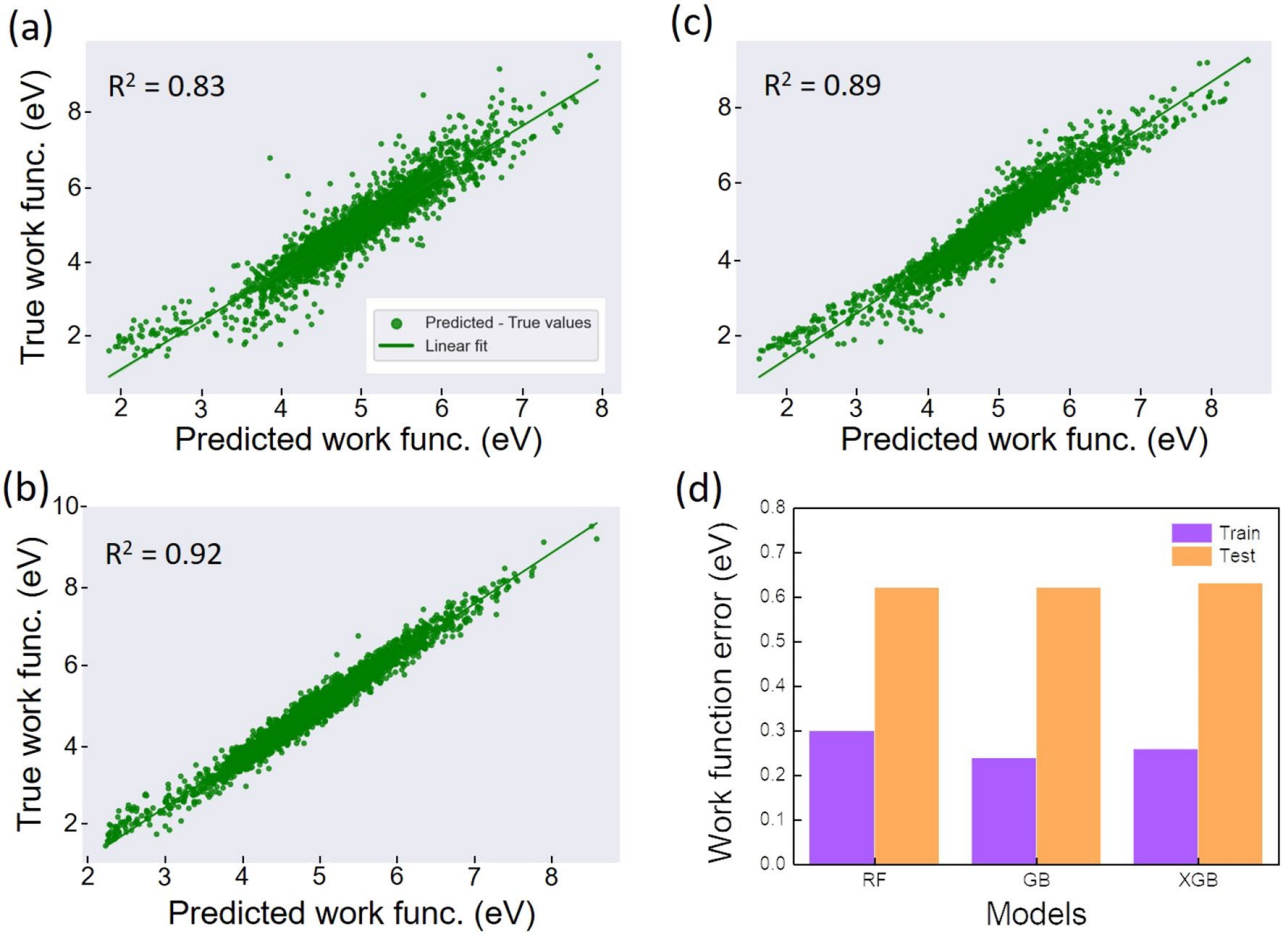
ML prediction of the work function with the feature set 1. Visualization of the predicted and true values of the work function (training set) obtained with RF, GB, XGB models (**a**,**b**,**c**). Bar plot shows the MAE errors of the training and test sets (**d**).

**Figure 5:**
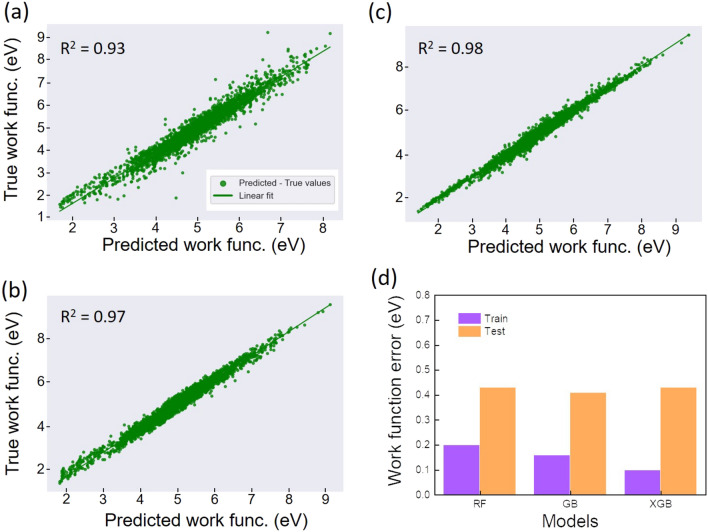
ML prediction of the work function with the feature set 2. Visualization of the predicted and true values of the work function (training set) obtained with RF, GB, XGB models (**a**,**b**,**c**). Bar plot of the MAE errors of the training and test sets (**d**).

We first consider the set of DB-based features (set 1). We find that the work function was predicted with high accuracy (R$^{2}>$ 0.8 and MAE about 0.2–0.3 eV) for the training dataset (Fig. 4). The test dataset shows an overfitting for both 3 models as observed previously (Fig. 4d). The MAE scores of the test dataset are around 0.62 eV which is relatively high compared to those of the training set (0.26 eV). The overfitting is expected due to the small scale of the dataset as aforementioned. The discrepancy between the training and test MAE values are found quite high, especially for GB model which shows the highest variation of 0.38 eV despite its R$^{2}$ value of 0.92. This means that the models, especially GB model, overfit the training set and catch much noise of the data. The results of the additional features are shown in Fig. 5. The training models are greatly improved in terms of R$^{2}$ and MAE scores. This finding indicates a booster effect of the additional features on the training process, which evidences a correlation between the generated features and the predicted property. A closer inspection on the results points out that R$^{2}$ value reaches the highest value of 0.98 for XGB model with a MAE value of 0.10 eV. Likewise the bandgap prediction, the training of the GB-based algorithm seems to couple quite well with the generated features, leading to an out-performance of the model on the training set.

The performance of the models on the test set also shows the same trend: the MAE values generated by the trained models are decreased down to 0.43 eV (Fig. 5d). Interestingly, it turns out that the improvement of the outcome metrics is associated with a noticeable discrepancy of MAE values between the training and test sets. This suggests that the additional features contributed to attenuate the overfitting effect. Indeed, we find that the average discrepancy is reduced of about 0.1 eV which is non negligible. Note that such a report of overfitting, which is necessary for ML processes was not mentioned in the previous studies on property predictions^15,20^. For a comprehensive assessment of the predicted values, we further calculate the mean absolute percent error (MAPE) which is also an accuracy indicator of the model performance on the test set. All MAPE values generated by the models are less than 10% (9.4%, 9.1% and 8.9% for RF, GB and XGB, respectively). This means that the models are stable and properly perform on the test dataset. These results unveil the fact that together with empirical function-based property, descriptor engineering in the framework of vector-based features greatly improves the model training and its performance on the unseen data. Therefore, the results allow to validate our approach in processing material data inputs/features in the ML training and data-driven predictions.

**Figure 6:**
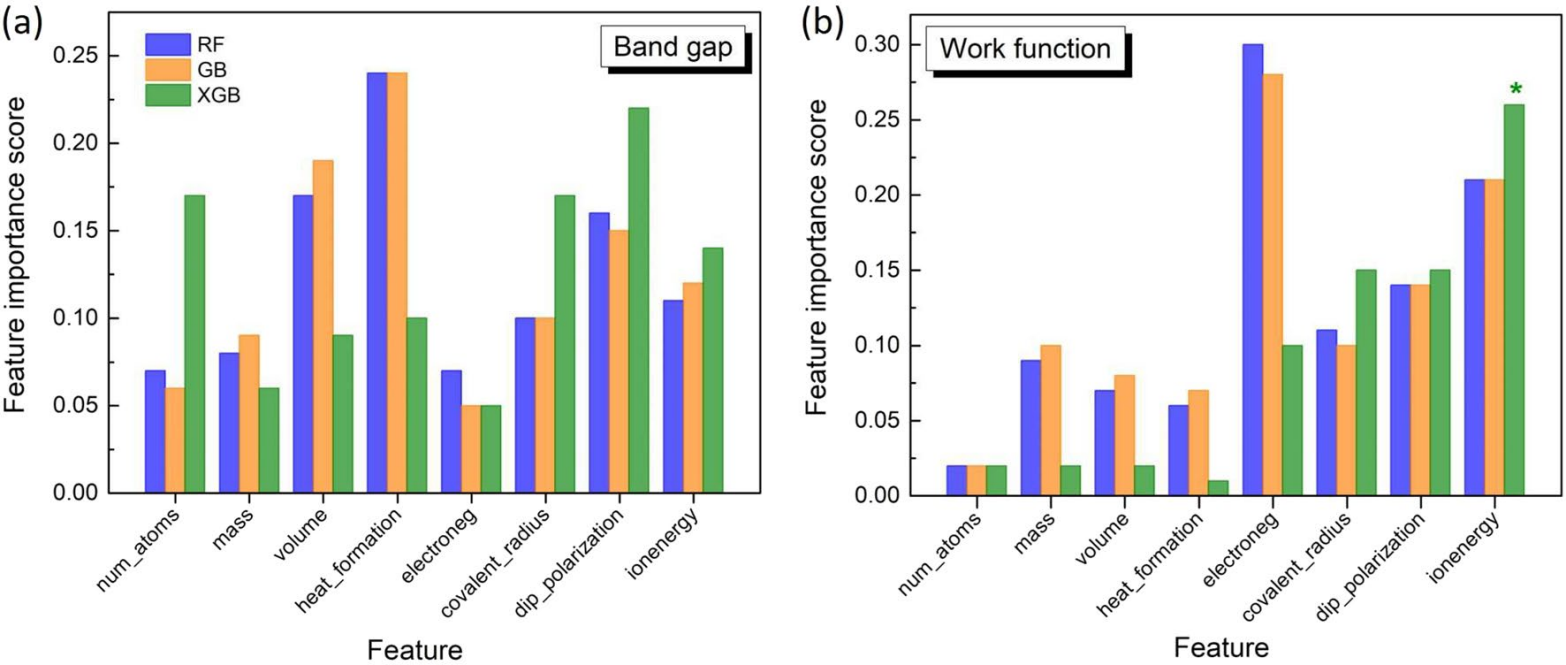
Feature importance scores of the input features for RF, GB, XGB models. The scores obtained from the bandgap prediction (**a**) and the work-function prediction (**b**). In (**b**) the asterisk (*) means that the magnitude of “ionenergy” for XGB model was divided by 2.

### Discussion

The feature importance regarding bandgap and work-function predictions shown in Fig. 6 corroborates the merit of additional features generated by property vectors. The block scores of these features (set 2) are quite high as compared to those of DB-based features (set 1) for both band gap and work function. In particular, the additional features completely dominate the ML prediction of the work function with an extremely high score of the feature “ionization energy” for XGB model. As a whole, the score importance of the additional features remains fairly stable when we change the property targets and ML algorithms while that of the DB-based features is not homogeneously distributed, indicating a strong impact of the additional features on the predicted properties. This points out to an universal characteristic of our generated features regarding the prediction of electronic structure properties for 2D materials. We stress that the construction of the generated features based on elemental properties is simple and low-computation cost. The generated features are strongly connected to electronic structure properties of 2D materials *via* atomic interactions. This relationship is one of the main factors that helps the ensemble ML models to learn efficiently. As the prediction of electronic structure properties is a supervised regression problem, the results in this study have shown that ensemble models appear to be appropriate for this purpose. Indeed, averaging (RF) or boosting (GB, XGB) approaches from individual learners allowed to efficiently reduce the error of the predicted values compared to the observed values. The choice of algorithms is crucial for mapping the relationship between features and target properties and RF, GB, XGB models have done this task successfully. The splitting mechanism at tree nodes of the ensemble ML algorithms using least-mean-square optimizer greatly fits the non-linearity relationship between the features and the electronic structure properties. Such a monotonous relationship can be pre-evaluated thanks to rank’s correlation (Fig. S3, Supplementary Information). As a matter of fact, multilayer perceptron-based model, a simple neural network, in which a subsequent propagation of nodes in training process is rooted to a linear relationship : ${\hat{y}} = \Phi (b_{i} + \sum _{i=1} x_{i}\omega _{i})$, where $\Phi$ is activation function, $b_{i}$ is bias at node i, $x_{i}$, $\omega _{i}$ are values and weights of adjacent nodes connected to node i, showed a very poor training and prediction with the additional features designed in this study. Indeed, we found R$^{2}$ = 0.46 and 0.79 with MAE = 0.56 eV and 0.36 eV for band gap, work-function predictions on training set, respectively.

### Relevance of the descriptor engineering-based ML prediction

**Figure 7:**
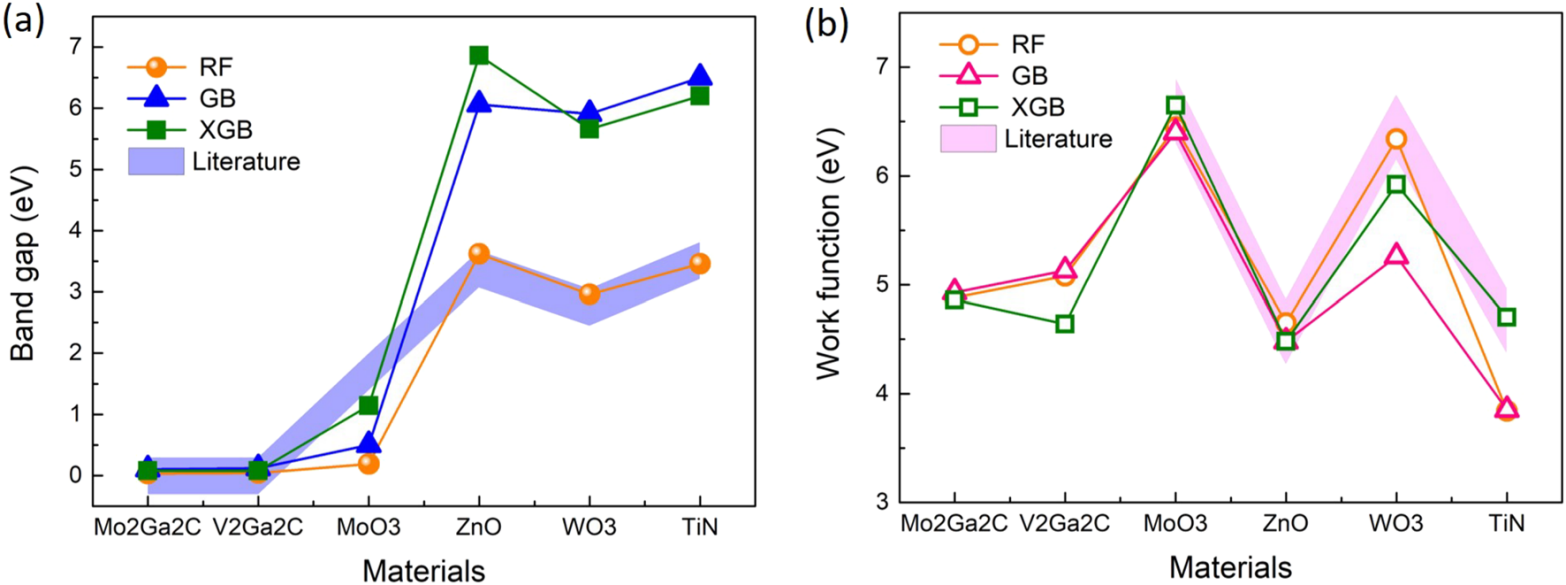
Prediction outcomes of the trained models (RF, GB, XGB) with help of descriptor engineering on selected 2D materials that are not available in the C2DB dataset. The filled areas [violet for band gap (**a**) and magenta for work function (**b**)] represent values collected in the literature within the error range of 0.3 eV.

Carbide-based, oxide-based and nitride-based 2D materials are new classes of 2D materials that have drawn increasing attention thanks to their outstanding electrical and opto-electrical properties. To demonstrate the practical use and the relevance of our ML approach as compared to costly computation techniques, we take a couple of 2D materials (experimentally known or not yet) belonging to these classes of materials those data are not available in the C2DB DB. $\hbox {Mo}_{2} \hbox {Ga}_{2}$C and $\hbox {V}_{2} \hbox {Ga}_{2}$C belong to 2D carbides like transition metal carbo-chalcogenides (TMCC) which are successfully synthesized recently^34^. $\hbox {Mo}_{2} \hbox {Ga}_{2}$C and $\hbox {V}_{2} \hbox {Ga}_{2}$C possess metallic character and laminated structure which may be used as MAX phase, a parent material for MXenes synthesis^35^. Only $\hbox {Mo}_{2} \hbox {Ga}_{2}$C thin film has been synthesized and reported^36^. The 2D oxides and nitride exhibit a semiconductor behaviour with large band gap according to theoretical and experimental works^37–41^. The band structures of 2D oxides and nitride show great potential in photonic, electronic and catalysis applications. Figure 7 shows the bandgap and work-function predictions of the two types of 2D materials (conductors and semiconductors). The filled areas depict the values of band gap and work function of these materials reported in the literature and obtained mostly by costly computation^37–45^. We set an error range of 0.3 eV represented by the filled areas for a straightforward comparison with our ML approach. To our knowledge, no data of work function for $\hbox {Mo}_{2} \hbox {Ga}_{2}$C and $\hbox {V}_{2} \hbox {Ga}_{2}$C are available.

Regarding the bandgap prediction, the three models predict quasi-zero band gaps for $\hbox {Mo}_{2} \hbox {Ga}_{2}$C and $\hbox {V}_{2} \hbox {Ga}_{2}$C, which is in good agreement with the literature. For greater values of band gap, the models could catch the variation tendency. However, GB and XGB models poorly predict the values of 2D oxides and nitride while RF performs quite well the prediction for these materials except for $\hbox {MoO}_{3}$. We anticipate that GB, XGB are likely sensitive to a poor distribution of the training dataset with respect to this range of bandgap values (typically 3 - 4 eV) as shown in the band gap histogram of the dataset (Fig. S2, Supplementary Information). The data concerning $\hbox {MoO}_{3}$ we collected are perhaps not reliable for inference. For the work-function prediction, the three models infer really well the trend of the work function in 2D oxides and nitride. Interestingly, the XGB model nicely predicts the values of all 2D oxides and nitride in the error range of 0.3 eV compared to those of the literature. The relative errors of the band gap and the work function are calculated and can be found in the section 7 of Supplementary Information. Finally, we anticipate values of 4.9 eV and 4.6 eV for the work functions of $\hbox {Mo}_{2} \hbox {Ga}_{2}$C and $\hbox {V}_{2} \hbox {Ga}_{2}$C, respectively.

## Conclusion

To sum up, a hybrid descriptor handling based on vectorized property matrices and molecular empirical function was proposed to train models and infer the electronic structure properties of 2D materials. We have shown that the data processing for building the descriptors is straightforwardly accessible, low-resource and highly efficient for ML training/inference. We have employed the four basic features (set 1) offered by the C2DB DB as a reference. The data-driven outcome obtained from these features indicates a fair performance of ML models, which reflects a weak relationship between the features and the band gap. Indeed, the ensemble models used to perform the prediction on the band gap show low values of R$^{2}$ ($\ge$ 0.58) and decent errors (MAE up to 0.43 eV). When we integrate the generated features into the ML process, the models show a great improvement with highest R$^{2}$ = 0.95 for XGB model. The MAE errors are substantially reduced down to maximum of 0.23 eV. The feature selection and feature extraction by the method described in this study have confirmed a strong relationship between the generated features with the band gap. An application of the method on the prediction of the work function, another electronic structure property, has been carried out. Similar tendency was observed with R$^{2}$ $\ge$ 0.9 and low MAE values. Furthermore, during the ML process, we are aware of the existence of overfitting, which is characterized by high error metrics of the ML performance on the test set with respect to the training set. The training involving the additional features contributes to attenuate this effect (about 0.1 eV). Finally, the inference of the band gap and the work function on new classes of 2D materials (carbides, oxides, nitrides) using the descriptor-tailoring method have revealed a decent correlation between the outcome values and those of the literature (*ab initio* computations). By considering both the ML metrics and the performance on the completely unseen data, RF and XGB models that are trained with C2DB data in the framework of our descriptor engineering are good models for predicting the band gap and the work function for 2D materials. The results derived from vector-based features combined with empirical features benchmark an alternative concept of descriptor engineering that is simple to implement, robust and relevant to tree-based ML models in terms of model selection, training and inference. This work provides feature template extendable to other properties for further study in ML-led applied physics with low-resource requirement in the framework of the predictive regression.

## Supplementary Information

Supplementary Information.

## Data Availability

The data and the code (https://gitlab.com/tuan2dplus/2dmt) that support the study are available upon reasonable request from the corresponding author (mtd@crhea.cnrs.fr).
