# Supplementary material for: Descriptor engineering in machine learning regression of electronic structure properties for 2D materials
Source: Sci Rep. 2023 Apr 3;13:5426. doi: 10.1038/s41598-023-31928-7 (PMC10070413; doi:10.1038/s41598-023-31928-7)
Supplement: Supplementary file 1 — Supplementary Information. [file 41598_2023_31928_MOESM1_ESM.pdf]

SUPPLEMENTARY MATERIAL

Descriptor engineering in machine learning regression of electronic structure properties for 2D materials

M. T. Dau, M. Al Khalfioui, A. Michon, A. Reserbat-Plantey, S. Vézian, and P. Boucaud

Université Côte d'Azur, CNRS, CRHEA, rue Bernard Grégory, 06560 Valbonne, France

1. Descriptors

Two categories of descriptors ("Origin" column) used in the study and their description are listed in the Table S1. The 2 sets of input features are built from these descriptors :

- Feature set 1 is composed of num_atoms, mass, volume, heat_formation
- Feature set 2 is composed of num_atoms, mass, volume, heat_formation, electroneg, covalent_radius, dip_polarization, (first) ionenergy

Descriptor	Origin	Description	Construction type
num_atoms	C2DB	Number of atoms	DB
mass	C2DB	Molecular mass	DB
volume	C2DB	Volume of unit cell	DB
heat_formation	C2DB	Heat of formation	DB
electroneg	generated	electronegativity	empirical function
covalent_radius	generated	Length of covalent bond	vectorized property
dip_polarization	generated	Dipole polarizability	vectorized property
ionenergy	generated	Ionization energy (1st level of ionization)	vectorized property

Table S1. Description of descriptors

2. Evaluation metrics

We use the coefficient of determination R^2 and mean absolute error in our work as the evaluation metrics. The root mean squared error (rmse), another common metrics for regression, was not be mentioned in the study for performance evaluation for the following reason. As the rmse is very sensitive to outliers, this metrics appears to be less relevant for evaluation due to large number of outliers (or out-of-range values). As shown in Figure S1, the number of outliers (for the point of view of machine learning process) in our dataset with respect to the band gap and work function is quite high: about 280 "outliers" for the band gap and 110 for work function.

The expression of R^2 and mae are as follows :

$$R^2 = 1 - \frac{\sum(y_i - \hat{y}_i)^2}{\sum(y_i - \bar{y}_i)^2}$$

$$MAE = \frac{1}{n}\sum_{i=1}^{n}|y_i - \hat{y}_i|$$

where y_i and $\hat{y}_i$ are the observed and predicted values, respectively; $\bar{y}_i$ is mean value of all observed values; n is the size of sample set.

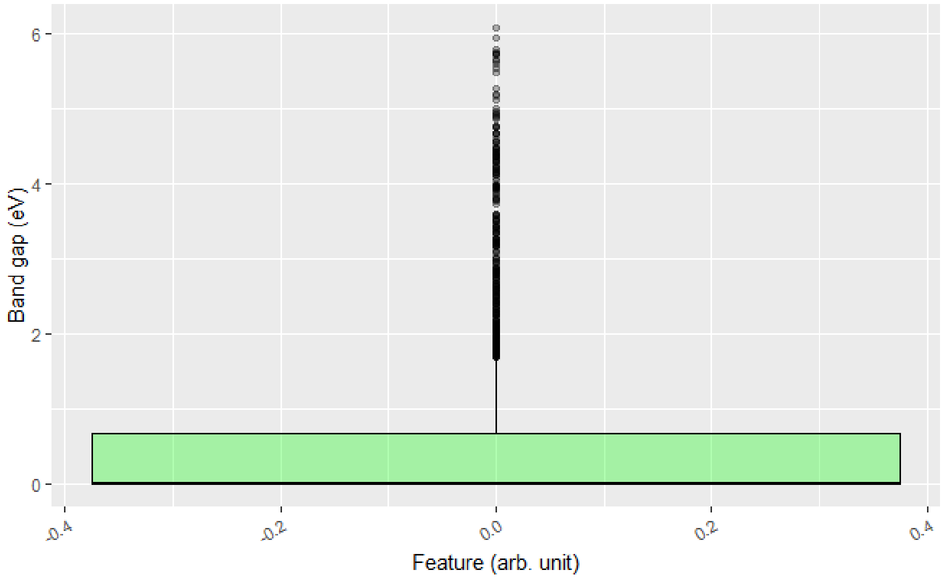

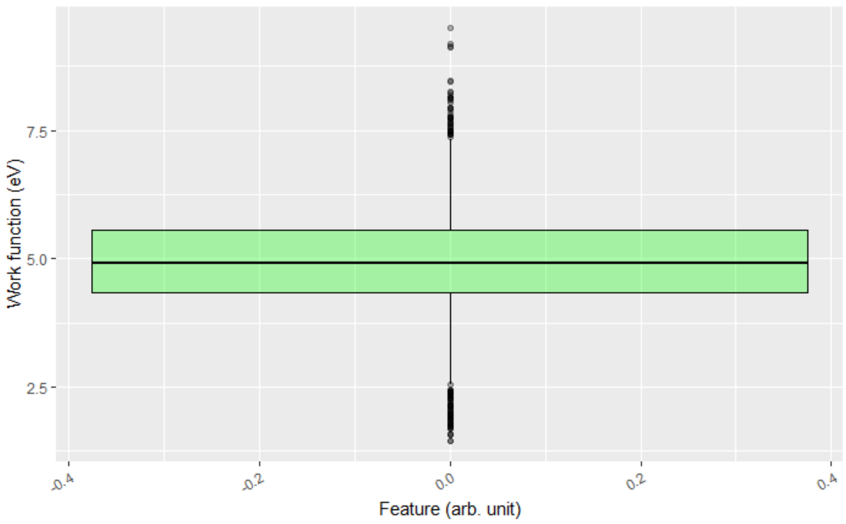

Figure S1. Box plot of band gap and work function displays location of minimum, median, first and third quartiles and maximum.

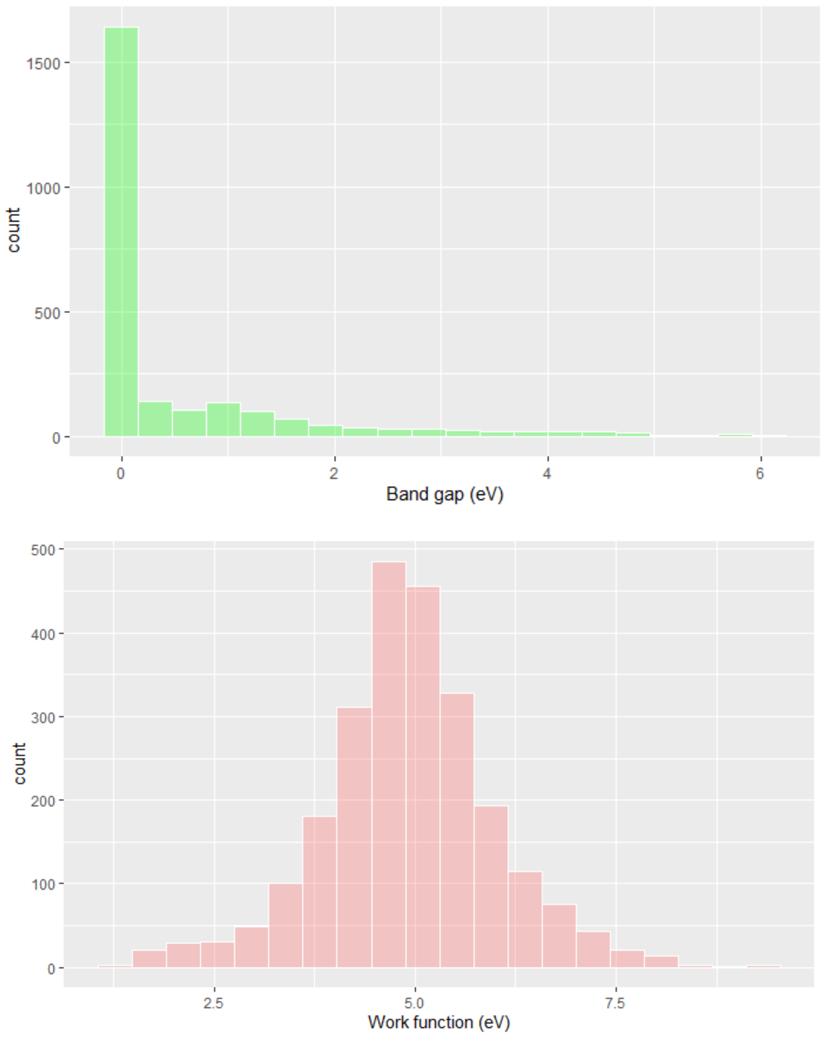

Figure S2. Histogram of band gap and work function extracted from the dataset

3. Grid search

Several relevant hyperparameters were tuned after having made some dry runs (pre-selection) in order to reduce the hyperparameter window size.

Random Forest model

```
n_estimators=[100, 200, 500, 1000, 5000],
min_samples_split=[3, 5, 7],
min_samples_leaf=[2, 3],
max_depth=[20, 40],
ccp_alpha=[0.0, 0.0001, 0.001],
max_features=[0.8, 1.0],
```

Gradient Boosting model

```
n_estimators=[100, 1000, 5000],
min_samples_split=[2, 5],
min_samples_leaf=[1, 2],
max_depth=[3, 21],
learning_rate=[0.001,0.01],
ccp_alpha=[0.0, 0.0001, 0.001],
max_features=[0.8, 1.0],
```

Extreme Gradient Boosting model

```
n_estimators=[100, 200, 500, 1000],
max_depth=[3, 6, 10],
eta=[0.3, 0.2, 0.1, 0.05, 0.02],
```

4. Feature correlation

A monotonous relationship between the features and target properties can be pre-evaluated by using the heatmap of Spearman correlation. Note that we use the first component of the vectorized property features for generating the heatmap.

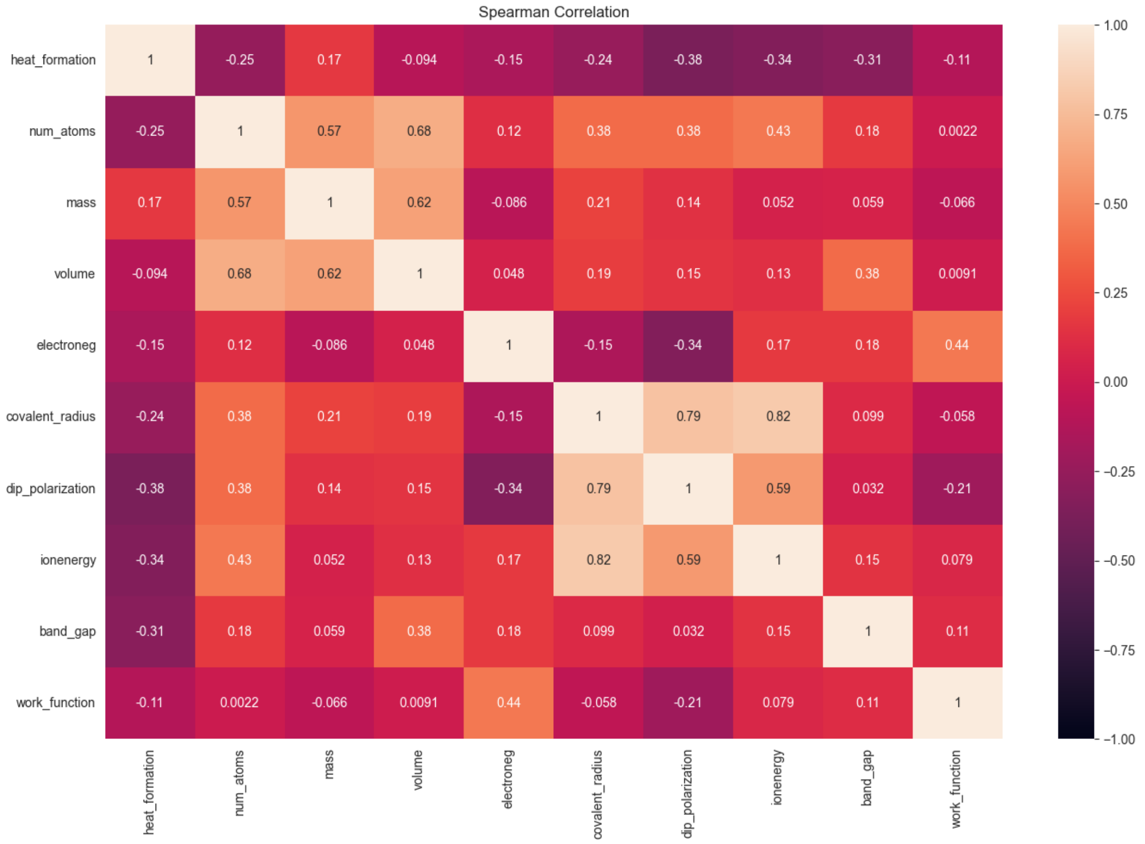

Figure S3. Heatmap of Spearman correlation of features and target properties

5. Model explanation

We use binary split (Classification And Regression Trees, CART framework) to explain the mechanism of prediction based on tree decision. The root node includes the whole dataset (y_i) which is split into parent (first split) and child nodes (second split) (Figure S4).

We explain here what is happening at one node (split and subset of samples). The tree growth is a recursive process for further nodes. The frame below is the common part for both random forest and gradient boosting.

The split algorithm follows regression tree's principle which is based on the threshold x_0 of the feature (feature 1, 2, 3 denoted as either atom number or volume, etc...). The instances (samples) having values of feature 1 > x_0 fall into the right parent node and others into the left parent node [1]. The weighted squared error is used (sum of 2 partitioning sub-sets) for computing the cost of each split (determination of x_0).

The predicted values $\hat{y}_i$ (predicted band gap or work function) at each node is the average of all samples ($\bar{y}$) in this node which permits to minimize the cost function.

$$L_{min} = \frac{1}{J}\sum_{i=1}^{J}[y_i - \hat{y}_i]^2 = \frac{1}{J}\sum_{i=1}^{J}[y_i - \bar{y}]^2 \quad (1)$$

where J stands for number of samples in the node and y_i is the observation values (band gap or work function values given by the database) of J samples in the node.

This is generalized until the terminal nodes (leaves). The output of each leave is the average of samples inside the leaf.

The tree growth will stop when the converging criterion is satisfied. This criterion is a tunable hyperparameter that is defined as max_depth, the maximum depth of tree or the length longest path from root to leaf.

In random forest, the final predicted value is the average of all predicted values generated by all the trees.

In gradient boosting model [2], $\hat{y}_i$ and $\bar{y}$ in equation (1) are replaced by residuals (r_{mi} , $\bar{r}_m$): $F_{m-1} + r_{mi}$ and $F_{m-1} + \bar{r}_m$. Here, F_{m-1}, r_{mi} and $\bar{r}_m$ denote the predicted value of previous tree (m-1), residuals at the considered node and the average of residuals of the node, respectively. The derivative of the loss function as a function of predicted value gives the (inverse) direction (gradient descent) in which the loss function is minimized. This direction corresponds to the residual of samples, hence, the name of gradient boosting model. The outcome of the tree growth in the gradient boosting is the updated predicted value $F_m(x) = F_{m-1}(x) + \delta \sum_{n=1}^{k} \bar{r}_{mn} I(x \in L_{mn})$ where x being sample, δ being learning rate (from 0 to 1), L_{mn} being terminal region (leaves), k being the number of terminal nodes (leaves), thus $\sum_{n=1}^{k} \bar{r}_{mn} I(x \in L_{mn})$ is the residual of the terminal node in which a sample x falls into. Extreme gradient boost adds a (customized) regularization term in the loss function helping to train the trees.

Initial predicted value $F_0 = \bar{y}$ for gradient boosting

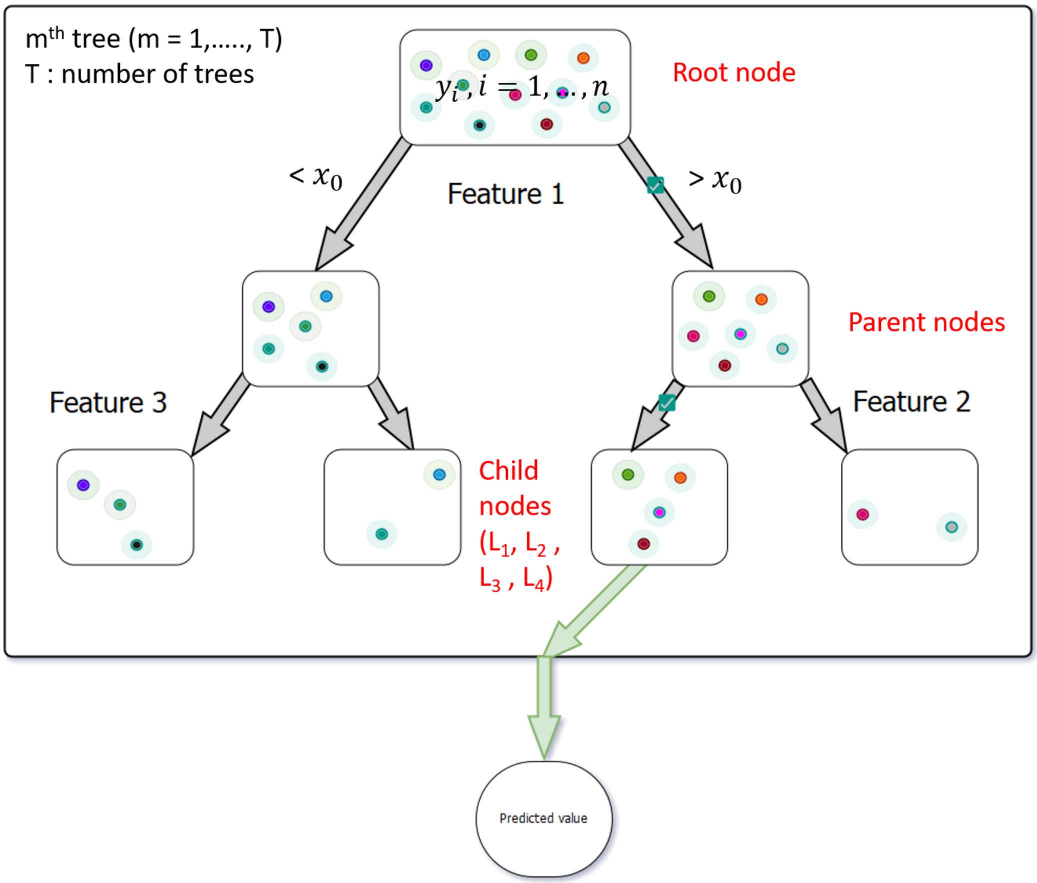

Figure S4. Schematics of the tree growth and feature-assisted split upon the training.

6. Dataset of selected 2D materials from different classes

Materials	heat_formation_evperatom	number_atoms	mass_au	volume_ang3
Mo_2Ga_2C	-0.009	5	343.34	170
V_2Ga_2C	-0.0006	5	253.34	154.3
MoO_3	-0.0104	4	143.96	215.35
ZnO	-3.632	2	81.38	49.72
WO_3	-8.735	4	231.84	57.63
TiN	-3.47	2	61.87	76.96

The heat enthalpies and the cell volumes of materials were collected from the references [3-5]

7. Relative errors of prediction on the 2D carbides, nitrides and oxides

Materials	Band gap			Work function		
	RF	GB	XGB	RF	GB	XGB
Mo_2Ga_2C	0.03	0.1	0.08	-	-	-
V_2Ga_2C	0.04	0.12	0.08	-	-	-
MoO_3	0.88	0.71	0.32	0.02	0.03	0.01
ZnO	0.07	0.79	1.03	0.01	0.02	0.02
WO_3	0.07	1.14	1.05	0.01	0.18	0.08
TiN	0.01	0.85	0.76	0.17	0.17	0.01

Values in red are absolute values (band gaps of conductors are zero).

References

[1] M. Krzywinski, N. Altman, *Nature Methods* **14**, 757 (2017)

[2] C. Bentéjac, A. Csörgő, G. Martínez-Muñoz
https://doi.org/10.48550/arXiv.1911.01914 (2019)

[3] A. Thore, M. Dahlqvist, B. Alling, J. Rosen
Phys. Chem. Chem. Phys. **18,** 12682 (2016)

[4] https://webbook.nist.gov/chemistry/form-ser

[5] https://materialsproject.org/
